# Registry of idiopathic inflammatory myopathies (REMAS) from nine Brazilian research centers linked to tertiary care and teaching hospitals

**DOI:** 10.1186/s42358-026-00522-6

**Published:** 2026-02-03

**Authors:** Fernando Henrique Carlos de Souza, Renata Miossi, Daniel Brito de Araujo, Jean Marcos de Souza, Simone Appenzeller, Luiz Felipe Adsuara de Sousa, Alexandre Moura dos Santos, Lorenza Rosa Silverio da Silva, Marlise Sitima Mendes Simões Faria, Juliana Almeida Vieira, Laurindo Ferreira da Rocha Junior, Luiz Samuel Gomes Machado, Ana Cecília Diniz Oliveira, Juliana Markus, Ana Gabriela da Silva, Flávia Patrícia Sena Teixeira Santos, Samuel Katsuyuki Shinjo

**Affiliations:** 1 Division of Rheumatology, Hospital das Clínicas HCFMUSP, Faculdade de Medicina, Universidade de São Paulo, Sao Paulo, SP Brazil; 2 Department of Internal Medicine, Universidade Federal de Pelotas (UFPel), Pelotas, RS Brazil; 3 Universidade Estadual de Campinas (UNICAMP), Campinas, SP Brazil; 4 Irmandade Santa Casa de Misericórdia de São Paulo, São Paulo, SP Brazil; 5 Universidade Federal de Pernambuco (UFPE), Recife, PE Brazil; 6 Universidade Federal de São Paulo (UNIFESP), São Paulo, SP Brazil; 7 Universidade Federal de Uberlândia (UFU), Uberlândia, MG Brazil; 8 Universidade Federal de Minas Gerais (UFMG), Belo Horizonte, MG Brazil; 9 Disciplina de Reumatologia, Faculdade de Medicina, Universidade de Sao Paulo, Av. Dr. Arnaldo, 455, 3° andar, sala 3192 - Cerqueira César, Sao Paulo, CEP: 01246 − 903 Brazil

**Keywords:** Autoimmune myopathies, Inflammatory myopathies, Myositis, Registry

## Abstract

**Objectives:**

Idiopathic inflammatory myopathies (IIMs) are a group of rare chronic inflammatory diseases that affect the muscle, skin, and other organs. There is a severe lack of evidence for current treatment protocols for IIMs. The rarity of these conditions requires multicenter collaboration is vital to facilitate studies of pathogenesis, demographic, clinical, laboratory manifestations, comorbidities, treatment, and disease outcomes from that research group we established a national registry (REMAS) that aims to improve knowledge, facilitate research and clinical trials, and ultimately improve outcomes for these patients.

**Methods:**

A Brazilian network of nine centers and a research group was established to include, since December 2022, consecutive patients with dermatomyositis (DM), clinically amyopathic DM (CADM), inclusion body myositis (IBM), polymyositis (PM), immune-mediated necrotizing myopathy (IMNM), and anti-synthetase syndrome (ASyS).

**Results:**

Currently, the study has recruited 284 patients, the majority were diagnosed with DM (53.1%), were female (77.1%), and Caucasian (44%). The median age was 51 years (IQR: 41–62), the median age at the disease onset was 43 years (IQR: 33–55), and the median duration of disease was 6 years (2–11). The median of the manual muscle test 8 (MMT-8) was 77 (74–80), the patient visual analogue scale (VAS) was 3 (0–5), physician VAS was 2 (0–5) and the Health Assessment Questionnaire of 0.33 (0.00–1.00), whereas median creatine kinase levels at the inclusion were 145 U/L (74–327). Among those positive for myositis-specific autoantibody, anti-Jo-1 was the most frequent (10.5%), followed by anti-Ro-52 (10.1%). Regarding treatment, 87% of the patients received prednisone with a median dose of 5 mg/day (0–20), 32.7% received pulse therapy with methylprednisolone, and 18.3% received human intravenous immunoglobulin.

**Conclusions:**

This establishment of this multicenter registry has facilitated research and advanced knowledge on a complex group of rare musculoskeletal diseases. The establishment of the REMAS has highlighted important local findings, despite limited representation from some regions of Brazil.

## Introduction

Idiopathic inflammatory myopathies (IIM), or systemic autoimmune myopathies (MAS), represent a rare and heterogeneous group of autoimmune disorders primarily characterized by the involvement of striated muscles [1]. These myopathies are classified as dermatomyositis (DM), clinically amyopathic DM (CADM), inclusion body myositis (IBM), immune-mediated necrotizing myopathy (IMNM), anti-synthetase syndrome (ASyS), and polymyositis (PM) [1–4].

Few registries have been established for adults with IIM [4–9]. For instance, the MYOVISION registry included 9,049 participants from the USA and Canada [5]. This study identified a significant reduction in physical function among patients with IIM compared to those with rheumatoid arthritis and the general USA population. Moreover, the risk factors associated with poor health-related quality of life (HRQoL) include a history of joint and lung involvement, treatment-resistant disease, and diagnosis of IBM [5].

A Spanish multicenter cohort study enrolled 479 patients with IIM [6]. The most prevalent clinical subgroups were PM (29%) and DM (22%), followed by overlap myositis (20.5%), juvenile myositis (18%), cancer-associated myositis (8%), IMNM (1%), and IBM (1%). During the follow-up period, 114 deaths were documented, with cancer (24%), infections (23%), and cardiovascular events (21%) as the primary causes.

The EuroMyositis Registry, an extensive multicenter cohort, analyzed 3,067 IIM cases from 11 countries [7]. It predominantly comprised DM cases (31%), highlighting the importance of extramuscular involvement in IIM patients, association with smoking in overlapping diseases, and convergence of lung involvement, dysphagia, malignancy, and cardiac involvement with disease severity. Malignancy was reported in 13% of cases and interstitial lung disease (ILD) in 30%, with the former more frequently associated with DM and the latter with ASyS.

The MYONET registry assessed 405 patients with DM and compared them with 649 patients with ASyS [8]. Within the ASyS cohort, 31% of the patients exhibited DM-like skin involvement (ASyS-DM skin). A higher prevalence of extramuscular manifestations, including mechanic’s hands, Raynaud’s phenomenon, arthritis, ILD, and cardiac involvement, distinguished ASyS with skin involvement from DM. Conversely, a higher incidence of four DM-like skin rashes differentiated DM from ASyS. Cancer-associated myositis was more prevalent in the DM cohort than in the ASyS cohort.

In South America, the Argentina registry [9], which includes 360 adults with IIM, has established a correlation with myositis-specific antibodies. Patients positive for anti-Jo-1, anti-Mi-2, and anti-MDA-5 were compared with a reference group negative for all myositis-specific autoantibodies. Anti-Jo-1 was the most frequent antibody and was associated with ILD, whereas anti-MDA-5 was associated with CADM and ILD, and anti-Mi-2 was associated with classic DM.

Considering the limited registries available in the literature, we present important demographic and epidemiological characterization from the nine Brazilian research centers that participated in the study.

## Patients and methods

This study represents a national observational prospective registry of patients with IIM (Registro Brasileiro de Miopatias Autoimunes Sistêmicas - REMAS). The nine participating centers in Brazil submitted the study protocol to their respective local ethics committees.

At each center, the local coordinator or designated representative assessed the patients as having IIM during routine clinical visits. Patients diagnosed with IIM and receiving regular outpatient care at the participating centers were consecutively recruited by rheumatologists. Eligible individuals were invited to participate in the registry and provided written informed consent before inclusion.

Diagnostic variables were documented, with inclusion criteria based on the European League Against Rheumatism/American College of Rheumatology (EULAR/ACR) 2017 classification criteria [10] for DM, CADM, IBM, and PM. These entities were categorized as clinical subtypes, independent of autoantibody profile or muscle biopsy findings. IMNM was defined according to the 224th European Neuromuscular Centre (ENMC) International Workshop criteria [11], and ASyS according to the Connors criteria [12].

Patients with other types of myopathies, such as muscular dystrophies and neuromuscular disorders, were excluded.

The following data were systematically collected:

- Demographic data: age at symptom and disease onset, age at inclusion in the registry, disease duration, sex, employment status, and ethnicity.
- Disease variables: Clinical symptoms (weight loss, fever, muscle weakness, and synovitis); cutaneous manifestations (heliotrope, Gottron’s papules, Gottron’s sign, mechanic’s hands);
- Exams (all when available): muscle biopsy; serum level of creatine kinase (CK); autoantibodies (antinuclear antibodies [ANA], myositis-specific and myositis-associated autoantibodies); electromyography results; chest computed tomography; lung function test results; capillaroscopy results; and muscle magnetic resonance imaging (MRI);
- Disease status at inclusion and follow-up visits, based on the: International Myositis Assessment and Clinical Studies Group (IMACS), which includes application of questionnaires and scores of the global assessment of disease activity perceived by the patient and physician using the visual analog scale (VAS), Manual Muscle Testing-8 (MMT-8), Health Assessment Questionnaire (HAQ), and extramuscular manifestations of the disease assessed by the Myositis Disease Activity Assessment Tool (MDAAT) [13–15]. MMT-8 evaluates the strength of eight muscle groups on the dominant side: shoulder abductors, elbow flexors, wrist extensors, hip flexors, knee extensors, ankle dorsiflexors, neck flexors, and hip abductors. Each muscle group is scored on a 0–10 scale, with higher scores indicating greater muscle strength, providing a standardized measure of overall muscle function [13–15];
- Treatment and treatment outcome (glucocorticoids and immunosuppressive drugs);
- Disease relapses;
- Habits: alcoholism, smoking, sedentary lifestyle;
- Comorbidities: arterial hypertension, diabetes mellitus, dyslipidemia, acute myocardial infarction, stroke, thyroid disease, fibromyalgia, depression, nephropathy, liver disease, and neoplasia;
- Infections: mild or severe (need for parenteral antibiotic therapy or hospital admission);
- Previous surgery;
- Gynecological (menstrual) and gestational history;
- Mortality.

Myositis-specific and myositis-associated autoantibodies. Blood samples (20 mL) were collected after a 12-hour fast, at inclusion in the study at the respective centers. The samples were coded and sent to the Inflammatory Myopathies Laboratory (Division of Rheumatology, Faculdade de Medicina, Universidade de São Paulo, SP) These were determined using a commercially available line blot test kit (Myositis Profile Euroline Blot test kit, Euroimmun, Lübeck, Germany), according to the manufacturer’s protocol, in which the following antibodies are tested: Anti-Jo-1, Anti-OJ, Anti-EJ, Anti-PL-7, Anti-PL-12, Anti-Zo, Anti-Ha, Anti-KS, Anti-NXP-2, Anti-TIF-1-𝛾, Anti-SAE, Anti-PM/Scl, Anti-MDA5, Anti-SRP, Anti-HMG-CoA-reductase, Anti-Mi-2, Anti-Ro52, and Anti-Ku. Antinuclear antibodies were detected by indirect immunofluorescence using HEp-2 cells as the substrate.

Data collection. Study data were collected and managed using Research Electronic Data Capture (REDCap) tools hosted at the Faculty of Medicine of the Universidade de São Paulo [16, 17] using a customized data collection model specifically designed for the REMAS registry. Each participating center had individual access credentials (login and password) to ensure data confidentiality and restricted access. The entire data management process complies with Brazilian legislation on data protection, ensuring ethical handling, storage, and privacy of personal and sensitive information [18].

Disease duration before treatment, treatment duration, and follow-up period for each patient were not collected in the present study.

Statistical analysis. No statistical analysis was performed, as the study was purely descriptive. Continuous data are presented as medians and interquartile ranges (IQR, 25th–75th), unless otherwise specified, whereas categorical data are presented as frequencies (%).

## Results

In this study, a cohort of 284 patients diagnosed with IIM was analyzed. The analysis covered the period from December 2022 to October 2024, with the majority of patients (66.1%) originating from the State of São Paulo. The cohort comprised predominantly female patients (77.1%), and most patients were identified as White(44%). Only 42.6% of the patients were economically active (Table 1).

**Table 1:** Demographic, clinical, and laboratory findings in 284 Brazilian patients with idiopathic inflammatory myopathies

Median of age (years)	51 (41–62)*
Median of disease duration (years)	6 (2–11)
Female	219 (77.1)
White ethnicity	125 (44.0)
Economically active	121 (42.6)
DM (%)	151 (53.1)
ASyS (%)	65 (23.1)
CADM (%)	27 (9.8)
PM (%)	26 (9.1)
IMNM (%)	12 (4.2)
IBM (%)	3 (1)
Skin lesions	
Heliotrope rash	108 (38)
Gottron’s signal	91 (32)
Gottron’s papules	108 (38)
Shawl sign	58 (20.4)
“V” neck sign	57 (20.0)
Mechanic’s hands	57 (20.0)
Articular, n (%)	102 (36)
MMT-8 (0–80)	77 (74–80)*
Patient’s VAS (0–10)	3 (0–5)*
Physician’s VAS (0–10)	2 (0–5)*
HAQ (0.00–3.00)	0.33 (0–1.00)*
Muscle enzymes	
Median creatine phosphokinase (U/L)	145 (74–327)*
Up 2x ULN	104 (36.6)†
2–4 x ULN	43 (15.1)†
4-6x ULN	30 (10.5)†
>6x ULN	105 (36.9)†
Positive antibodies	92 (37.2)**
Anti-Jo-1	26 (10.5)
Anti-Ro-52	25 (10.1)
Anti-MDA 5	12 (4.9)
Anti-PM/Scl	3 (1.2)
Anti-Mi-2	4 (1.6)
Anti-SAE	1 (0.4)
Anti-TIF-1 g	4 (1.6)
Anti-SRP	1 (0.4)
Anti-Ku	0
Anti-PL-7	5 (2.0)
Anti-PL-12	1 (0.4)
Anti-EJ	2 (0.8)
Anti-NXP-2	0
Negative antibodies	
DM(%)	96 (76.8)
CADM (%)	16 (64.0)
IBM (%)	1 (33.3)
IMNM (%)	7 (77.7)
PM (%)	16 (76.1)
ASyS (%)	19 (29.6)
Not tested	
DM(%)	26 (17.2)
CADM (%)	3 (10.7)
IMNM (%)	3 (25.0)
PM (%)	5 (19.2)

Data are expressed as median (25th − 75th), of frequency (%)

ASyS: Anti−synthetase syndrome; CADM: Clinically amyopathic dermatomyositis; DM: Dermatomyositis; HAQ: Health Assessment Questionnaire; IMNM: Immune−mediated necrotizing myopathy; IBM: Inclusion body myositis; MMT−8: Manual Muscle Testing−8; PM: Polymyositis; ULN: upper limit of normal; VAS: Visual analog scale

* Values at inclusion

** 247 (87%) patients tested

† Values at diagnosis

The median age of the patients was 51 (41–62) years, with a median age at disease onset of 43 (33–55) years, and a median disease duration of 6 (2–11) years (Table 1). The general clinical features are provided in Table 1.

The overall comorbidities and lifestyle habits are detailed in Table 2, with a notably high prevalence of sedentary lifestyles (83.8%). Among the clinical subtypes, 151 (53.1%) were diagnosed as DM, followed by ASyS (23.2%), CADM (9.8%), PM (9.1%), IMNM (4.2%), and IBM (1.0%).

**Table 2:** Associated comorbidities in 284 Brazilian patients with idiopathic inflammatory myopathies

Sedentary lifestyle	238 (83.8)
Systemic arterial hypertension	98 (38.1)
Obesity	71 (25.0)
Dyslipidemia	72 (25.1)
Diabetes mellitus	48 (16.7)
Hypothyroidism	33 (11.5)
Depression	32 (11.1)
Alcoholism	9 (3.1)
Smoking	14 (4.9)

^Data are expressed as frequency (%)^

Regarding disease status, the median MMT-8 score was 77 (74–80), whereas the patient and physician VAS scores were 3 (0–5) and 2 (0–5), respectively. The HAQ score was 0.33 (0.00–1.00), and the median CK level at inclusion was 145.0 (73.5-327.3) U/L (Table 1). At diagnosis, most patients exhibited elevated CK levels, with equal proportions of patients having levels twice the upper limit of normal (ULN) and above six times the ULN (37% each).

The majority (87%) of patients underwent autoantibody testing, with 155 (63%) testing negative. Among those who tested positive, anti-Jo-1 was the most frequently detected antibody (10.5%), followed by anti-Ro-52 (10.1%). The other autoantibodies tested are shown in Table 1 and a distribution of antibodies according to the clinical subtype is shown in Table 3.

**Table 3:** Myositis antibodies profile according to the clinical subtype

Antibody	Clinical subtype					
	DM	CADM	IBM	IMNM	PM	ASyS
Jo-1	1	0	0	0	1	24
PL-7	1	0	0	0	0	4
PL-12	0	0	0	0	0	1
EJ	0	0	1	0	0	1
OJ	0	0	1	0	0	0
Ks	0	0	0	0	0	0
Zo	0	0	0	0	0	0
YRS	0	0	0	0	0	0
Mi-2	2	1	0	0	1	0
PM/Scl	1	0	0	0	0	2
MDA5	5	7	0	0	0	0
SRP	0	0	0	1	0	0
HMGR	0	0	0	1	0	0
Ro-52	10	1	0	0	2	12
RNP	4	0	0	0	1	1
NXP-2	0	0	0	0	0	0
TIF-1 g	4	0	0	0	0	0
Ku	0	0	0	0	0	0
SAE	1	0	0	0	0	0

^ASyS: anti−synthetase syndrome; CADM: clinically amyopathic dermatomyositis; DM: dermatomyositis; IBM: inclusion body myositis; IMNM: immune−mediated necrotizing myopathy; PM: polymyositis^

A total of 150 (53%) patients underwent electroneuromyography, with 115 (77.0%) exhibiting a myopathic pattern. Additionally, 62 (21.8%) patients underwent muscle biopsy, and of these, 60% presented with perimysial inflammatory infiltration and/or perifascicular atrophy, findings more closely related to DM. In addition, 83 patients (29.2%) underwent muscle MRI, with 55 (66.3%) showing edema and 35 (42.2%) showing fatty replacement. Finally, 87 (31%) subjects underwent nailfold capillaroscopy, with 59 (68%) displaying a scleroderma (SD) pattern.

Regarding treatment, 86.6% of the patients received prednisone, with a median dose at inclusion of 5 (0–20) mg/day. Additionally, 32.7% of the patients received intravenous methylprednisolone and 18.3% received intravenous human immunoglobulin (Table 4). Various corticosteroid-sparing immunosuppressive agents were employed as monotherapy or in combination, depending on tolerance, side effects, and disease refractoriness. Azathioprine, methotrexate, and hydroxycholoroquine were used in 47.5%, 53.5%, and 26.1% of the cases, respectively (Table 4). At the time of inclusion in the present study, 38% of the patients were receiving a combination of two or more drugs of classical synthetic, targeted synthetic, and biological immunosuppressives.

**Table 4:** Drug therapy used in 284 Brazilian patients with idiopathic inflammatory myopathies

Drugs	*N* (%)
Methylprednisolone	93 (32.7)
Prednisone	246 (86.6)
Azathioprine	135 (47.5)
Methotrexate	152 (53.5)
Hydroxychloroquine	74 (26.1)
Mycophenolate mofetil	54 (19.0)
Cyclosporine	56 (19.7)
Leflunomide	22 (7.7)
Rituximab	36 (12.7)
Human intravenous immunoglobulin Combined therapy	52 (18.3) 27 (38.0)

^Data are expressed as frequency (%)^

Eighteen (6%) of patients had cancer. Among them, eight developed cancers prior to the diagnosis of myopathy, with the latter occurring after a median interval of 13 years. Ten patients developed cancer after myositis, with a median interval of two years between the two diagnoses. The identified malignancies included five breast cancers, four lymphomas, two thyroid cancers, two cervical cancers, two basal cell carcinomas of the skin, one nasopharyngeal squamous cell carcinoma, one bladder carcinoma, and one pancreatic carcinoma.

## Discussion

The REMAS constitutes the observational initiative to date, focused on characterizing patients with IIM in Brazil. Our study offers significant epidemiological and clinical insights into a population characterized by a high degree of miscegenation, which has been underrepresented in prior international registries. The predominance of DM and ASyS in our cohort aligns with the distribution observed in other large-scale registries, such as EuroMyositis and MYONET [7, 8]. However, variations in autoantibody profiles and disease presentation underscore the potential regional and ethnic influences on disease expression. When directly comparing REMAS to other major registries, such as EuroMyositis, MYONET, MYOVISION, and the Spanish registry, important differences emerge (Table 5). REMAS demonstrated the highest proportion of DM (53.1%) and seronegative patients (63%), along with a lower prevalence of PM (9.1%) and cancer-associated myositis (6%) relative to these international cohorts. The lower frequency of muscle biopsy and MRI use also reflects structural disparities in access to diagnostic tools across countries.

**Table 5:** Comparison of REMAS with major international IIM registries

	REMAS	EuroMyositis	MYONET	MYOVISION	Spanish Registry
Number of patients	284	~ 3,000	1,196	1,06	345
Median age (years)	51	53–56	~ 53	54	53
Female (%)	77.4%	~ 69%	~ 71%	~ 70%	~ 72%
Most frequent subtype	DM (53.1%)	PM (33%), DM (31%)	PM (~ 35%)	PM (~ 40%)	PM (42%), DM (22%)
Polymyositis	9.1%	33%	35%	40%	42%
IMNM	4.2%	NS	~ 5%	NS	NS
Anti-Jo-1	10.5%	~ 18%	20–25%	24%	22%
Seronegative	63%	NS	~ 35%	~ 30%	~ 38%
ILD	24%	~ 20%	28%	~ 25%	20–25%
Malignancy	6%	13–17%	14%	~ 10%	12%
Muscle biopsy	22%	~ 40%	> 50%	60%	75%
Corticosteroid use	87%	> 80%	~ 90%	~ 85%	~ 80%
Methotrexate use	53.5%	40–50%	45%	~ 50%	~ 48%
Azathioprine use	47.5%	~ 30%	~ 35%	28%	26%
IVIG use	18.3%	12%	~ 15%	~ 10%	NS

^DM: dermatomyositis; ILD: interstitial lung disease; IMNM: immune−mediated necrotizing myopathy; IVIG: intravenous immunoglobulin; NS: not specified; PM: polymyositis^

In our cohort, DM accounted for 53.1% of cases, a proportion exceeding that reported in the Spanish (22%) and EuroMyositis (31%) registries [6, 7]. Conversely, IMNM was less prevalent (4.5%), which may reflect underdiagnosis due to limited access to advanced diagnostic tools, such as muscle biopsy and myositis-specific antibody panels. Notably, PM constituted less than 10% of our cases, a finding consistent with recent discussions questioning the distinctiveness and frequency of PM as a clinical entity in the context of evolving classification criteria (4).

The predominance of female sex (77.4%) in REMAS aligns with the female-to-male ratio observed in other cohorts (2–3:1), although the proportion appears slightly higher than those reported in MYOVISION and EuroMyositis [5, 7]. The median age of our patients was 51 years, which is consistent with global estimates [1–4], yet a notable proportion of these individuals were economically inactive, reflecting the significant impact of the disease on daily functioning and employment.

Muscle weakness was the most common presenting symptom, consistent with the classical manifestations of IIM. The median MMT-8 score at inclusion (77) and the relatively low HAQ (0.33) indicated that many patients were enrolled at later stages of their disease, with several already exhibiting partial disease control. However, the proportion of patients with elevated CK levels at diagnosis (63% with levels at least twice the upper limit of normal) and the significant percentage showing biopsy or MRI abnormalities at inclusion (70% with muscle edema and/or fatty replacement on MRI) underscore the ongoing challenge of early diagnosis and adequate disease control.

Autoantibody testing identified a significant proportion of seronegative patients (63%), with anti-Jo-1 as the most frequently detected autoantibody (10.5%), aligning with international data and closely associated with ILD, as documented in the Argentine and MYONET cohorts [8, 9]. Anti-Ro-52, which often co-occurs with other myositis-specific antibodies and is linked to extramuscular manifestations, was the second most prevalent antibody in our study population.

Extramuscular manifestations, including ILD, malignancy and dysphagia are well-established contributors to IIM morbidity. In the REMAS cohort, 6% of the patients had associated malignancies, with breast cancer and lymphoma being the most common. Although this percentage is lower than the 13%-17% malignancy rates observed in EuroMyositis and MYONET [7, 8], the temporal association of cancer diagnosis relative to myositis onset underscores the necessity for long-term vigilance in this population. Continued follow-up is essential to capture delayed-onset malignancies and better characterize cancer-associated myositis in our cohort.

The therapeutic patterns observed in our registry largely align with international trends, characterized by the prevalent use of corticosteroids (87.0%) and immunosuppressives, such as methotrexate (53.5%) and azathioprine (47.5%), with combination therapy employed in 38% of cases. Notably, 18.3% of the patients received intravenous immunoglobulin. These statistics highlight the variability in therapeutic strategies across centers and underscore the need for more standardized treatment algorithms.

This registry also provides valuable data on the diagnostic approaches for IIM. Electromyography was conducted in over half of the patients (53%), MRI in 29%, whereas muscle biopsy was available in only 22% - a limitation common to many real-world studies due to access issues. Furthermore, nailfold capillaroscopy revealed scleroderma-like patterns in 68% of those tested, reinforcing vascular involvement, particularly in DM, and the need for comprehensive evaluation.

It is important to note that IIM are rare disorders that are frequently underdiagnosed or misdiagnosed. Similarly, several international registries [4–9] have also reported relatively small patient numbers. Furthermore, the fragmentation of medical records within the public healthcare system limits access to comprehensive clinical information, which represents an additional challenge in patient inclusion and should be considered when interpreting registry data.

Despite its strengths, the REMAS has limitations. The inclusion of patients was based on a clinician report, potentially introducing a selection bias. Additionally, the centralization of autoantibody testing does not occur in real time, which may have influenced treatment decisions. In addition, only nine centers have participated in the registry so far, with many regions of Brazil still underrepresented; thus a collective effort is currently underway to include more centers. Although REMAS only included patients who met the classification criteria from reference centers for IIM, it is known that some diseases mimic myositis, are difficult to diagnose and some of these could be included inadvertently in this registry.

Another important limitation of the REMAS registry is the non-application of newer classification proposals derived from recent ENMC workshops for specific idiopathic inflammatory myopathy subtypes, including DM, IBM, and ASyS. Although these frameworks offer refined, antibody- and pathology-oriented disease definitions, the REMAS registry was designed as a multicenter, real-world observational cohort, encompassing centers with heterogeneous access to advanced immunological testing, muscle biopsy, and specialized imaging.

Therefore, the 2017 EULAR/ACR classification criteria were intentionally adopted as the primary framework for defining clinical subtypes, given their broad international validation, feasibility across diverse clinical settings, and continued widespread use in registry-based research. The lack of uniform availability of diagnostic resources across participating centers limited the consistent application of newer ENMC-based criteria and may have influenced the distribution of specific disease subgroups, particularly antibody-defined phenotypes. This should be considered when comparing REMAS data with future registries adopting newer classification systems.

Finally, the substantial proportion of seronegative patients prompts consideration of whether more sensitive or novel assays could enhance diagnostic accuracy.

## Conclusions

Nevertheless, REMAS constitutes a significant advancement in understanding the spectrum of IIM in Brazil. This highlights the necessity for ongoing efforts to standardize diagnostic methodologies, broaden access to advanced immunological testing, and assess long-term outcomes, including malignancy risk, quality of life, and treatment response. Future prospective follow-up is essential for evaluating disease progression, relapse rates, and the impact of treatment strategies on functional outcomes.

## Data Availability

Not applicable.

## Abbreviations

ANA: Antinuclear antibodies
ASyS: Anti-synthetase syndrome
CADM: Clinically amyopathic dermatomyositis
CK: Creatine kinase
DM: Dermatomyositis
ENMC: European Neuromuscular Centre
HAQ: Health Assessment Questionnaire
HRQoL: Health-related quality of life
IBM: Inclusion body myositis
IIM: Idiopathic inflammatory myopathies
ILD: Interstitial lung disease
IMACS: International Myositis Assessment and Clinical Studies Group
IMNM: Immune-mediated necrotizing myopathy
MAS: Systemic autoimmune myopathies
MDAAT: Extramuscular manifestations of the disease assessed by the Myositis Disease Activity Assessment Tool
MMT-8: Manual Muscle Testing-8
MRI: Magnetic resonance imaging
PM: Polymyositis
REDCap: Research Electronic Data Capture
REMAS: Brazilian registry of systemic autoimmune myopathies
VAS: Visual analog scale
